# Collectins and ficolins in neonatal health and disease

**DOI:** 10.3389/fimmu.2023.1328658

**Published:** 2023-12-19

**Authors:** Maciej Cedzyński, Anna S. Świerzko

**Affiliations:** Laboratory of Immunobiology of Infections, Institute of Medical Biology, Polish Academy of Sciences, Łódź, Poland

**Keywords:** collectin, ficolin, mannose-binding lectin (MBL), neonate, prematurity, perinatal infection, pulmonary surfactant, respiratory distress syndrome (RDS)

## Abstract

The immune system starts to develop early in embryogenesis. However, at birth it is still immature and associated with high susceptibility to infection. Adaptation to extrauterine conditions requires a balance between colonization with normal flora and protection from pathogens. Infections, oxidative stress and invasive therapeutic procedures may lead to transient organ dysfunction or permanent damage and perhaps even death. Newborns are primarily protected by innate immune mechanisms. Collectins (mannose-binding lectin, collectin-10, collectin-11, collectin-12, surfactant protein A, surfactant protein D) and ficolins (ficolin-1, ficolin-2, ficolin-3) are oligomeric, collagen-related defence lectins, involved in innate immune response. In this review, we discuss the structure, specificity, genetics and role of collectins and ficolins in neonatal health and disease. Their clinical associations (protective or pathogenic influence) depend on a variety of variables, including genetic polymorphisms, gestational age, method of delivery, and maternal/environmental microflora.

## 1 Collectins and ficolins

The immune system starts to develop early in embryogenesis but at birth is still very immature, especially in prematurely born neonates. Therefore premature babies in particular are highly susceptible to infection. Adaptation to extrauterine conditions requires keeping a balance between colonization with commensal flora and protection from pathogens. Infections, oxidative stress and invasive procedures used for saving lives may lead to transient organ dysfunction, their permanent damage or even fatal outcome [reviewed in (1–4)]. In this review, the role of two distinct but structurally and functionally related molecular families, collectins and ficolins, is discussed in the context of neonatal health and disease.

**Figure 1:**
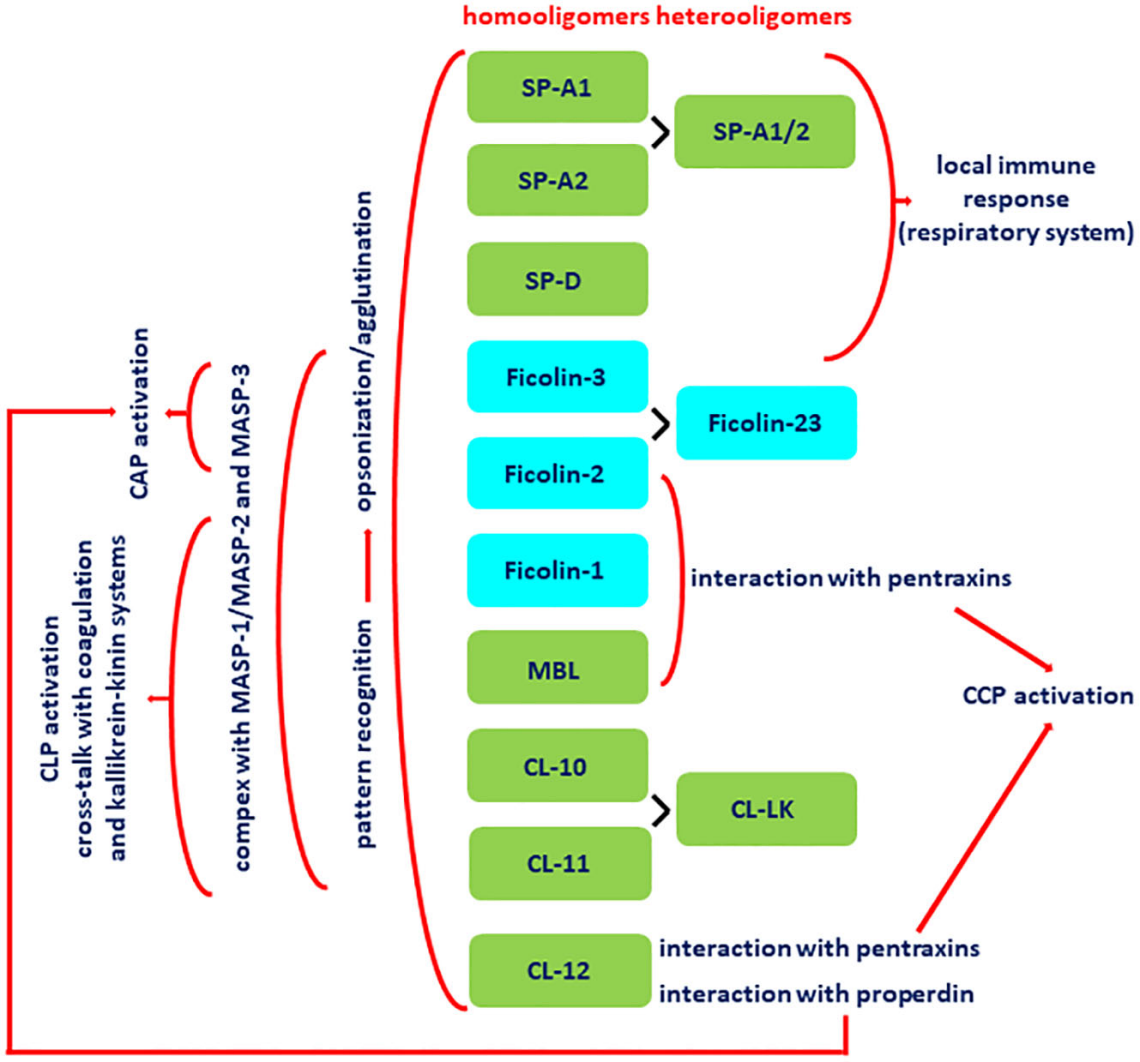
Basic activities, interactions and cross-talks of collectins (green boxes) and ficolins (blue boxes). All demonstrated collectins and ficolins are pattern-recognition molecules (PRM) able to opsonise and/or agglutinate pathogen cells. With an exception for SP-A and SP-D (pulmonary collectins) and CL-12, they form complexes with MASP-1, MASP-2 and MASP-3. Upon binding of PRM-MASP complex to the ligand, MASP-1 undergoes autoactivation and activates MASP-2, which substrates are C4 and C2 (that enables complement activation via the lectin pathway). Those enzymes contribute to cross-talk with coagulation system via cleavage of prothrombin (MASP-1 and -2) and factor XIII (MASP-1) as well as kallikrein-kinin system via cleavage of high molecular weight kininogen (HMWK) (MASP-1). MASP-3 substrate is pro-factor D (what enables contribution to complement activation via the alternative pathway). Collectin-12 due to interaction with pentraxins and properdin contributes to complement activation via classical and alternative pathways, respectively. MBL, ficolin-2 and ficolin-3 interacting with pentraxins are able to participate in complement activation via classical pathway. Pulmonary surfactant collectins and ficolin-3 are expressed within respiratory system and therefore participate in local immune response. CAP, complement alternative pathway; CCP, complement classical pathway; CLP, complement lectin pathway; CRP, C-reactive protein; SP-A1/2, heterocomplex of SP-A1 and SP-A2; CL-LK, heterocomplex of CL-10 and CL-11; ficolin-23, heterocomplex of ficolin-2 and ficolin-3; MASP, MBL-associated serine proteases.

Both collectins and ficolins are oligomers of basic subunits, consisting of three polypeptide chains. Each chain possesses four domains: a N-terminal cysteine-rich domain, followed by a collagen-like sequence, an α-helical neck region and a functional (pattern recognition) domain at the C-terminus. The functional domain differs between collectins and ficolins: it is a globular carbohydrate-recognition (lectin) domain (CRD) in collectins and a fibrinogen-like (FBG) domain in ficolins. The collagen-like region includes repeating Gly-X-Y triplets (where X and Y correspond to any amino acids). Both collectins and ficolins act as pattern-recognition molecules (PRM). They are able to interact with and participate in elimination of a variety of pathogens or altered autologous cells, by recognising pathogen- or danger-associated molecular patterns (PAMP, DAMP), through opsonisation or agglutination and (for all ficolins and some collectins) complement activation. In general, their activity requires the presence of calcium cations [reviewed in (5–7)]. The schematic overview of their activities, interactions with other immune factors and cross-talks is given in **Figure 1**.

### 1.1 Structure, specificity and genetics

#### 1.1.1 Structure and specificity of collectins

The term “collectin” reflects the structure and function of proteins constituting a subfamily of C-type lectins: COLlagenLECTIN. Three human collectins, mannose-binding lectin (MBL) (or mannan-binding lectin), collectin-10 (CL-10) (or collectin-liver 1, CL-L1) and collectin-11 (CL-11) (or collectin-kidney 1, CL-K1), form complexes with enzymes called MASP (MBL-associated serine proteases) and share ability to initiate complement activation via the lectin pathway. Those collectins can also cross-talk with the alternative pathway of complement, the coagulation cascade and the kallikrein-kinin system (5–7) (**Figure 1**). Another member of this subfamily, collectin-12 (CL-12) (or collectin-placenta 1, CL-P1) is known to contribute to complement activation via the alternative (8, 9) and classical (10, 11) pathways, due to interaction with properdin and short (C-reactive protein, CRP; serum amyloid P, SAP) or long (pentraxin 3, PTX3) pentraxins, respectively. Furthermore, MBL interacts with SAP and PTX3, resulting in enhancement of opsonophagocytosis and/or amplification of complement activation via cross-talk between lectin and classical pathways (12). The two other collectins, surfactant protein A (SP-A) and surfactant protein D (SP-D) are unable to activate complement directly or indirectly (13) (**Figure 1**).

**Figure 2:**
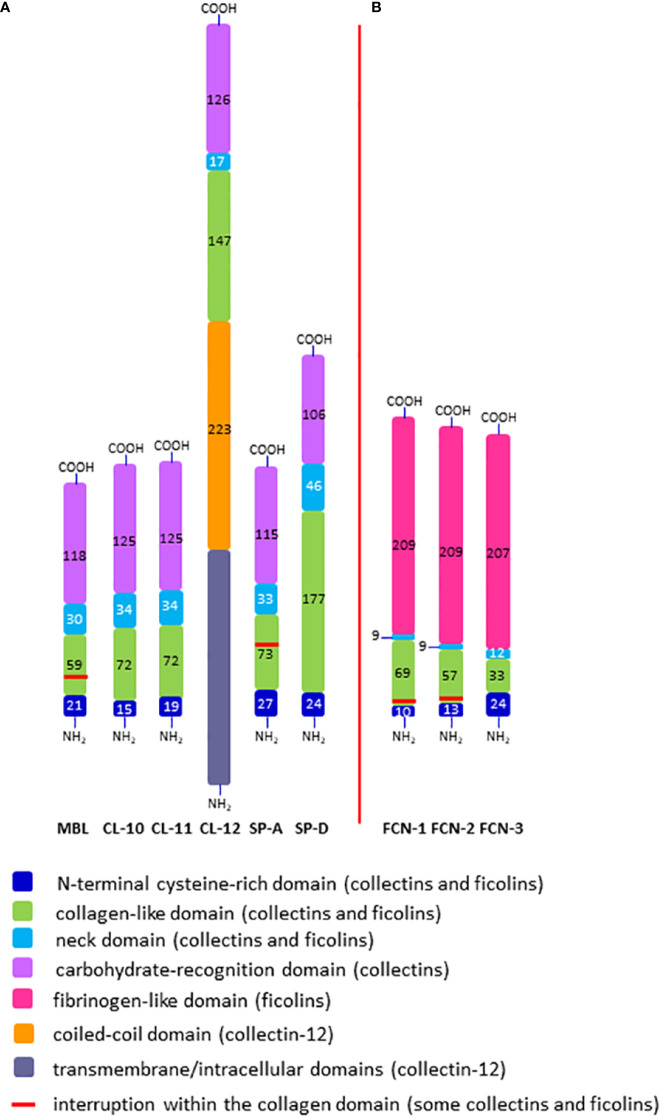
Domain organisation of polypeptide chains of collectins **(A)** and ficolins **(B)**. The length of each schematic domain is proportional to the number of amino acid residues (no secondary, tertiary or quaternary structures like α-helices, globular heads, trimers, multimers, *etc.* are shown). For collectin-12, details in ectodomain (including lectin and collagen-like domains) are presented while transmembrane/intracellular domains are displayed as one distinct region. SP-A corresponds to SP-A1 and SP-A2; FCN-1, -2, -3 correspond to ficolin-1, ficolin-2 and ficolin-3, respectively; -NH_2_ and -COOH correspond to the N- and C- termini, respectively.

The single polypeptide (glycosylated and acetylated) chain of MBL (32 kDa) consists of 228 amino acid (AA) residues: 21 (including 3 Cys) in the cysteine-rich domain, 59 in the collagen-like region (including 19 Gly-X-Y triplet repeats, interrupted after 7^th^ one by Gly-Gln), 30 in the neck domain and 118 in the CRD (**Table 1**; **Figure 2A**). The interruption gives rise to a kink that causes the “tulip bouquet” shape of the oligomer (up to hexamer) of subunits [reviewed in (6, 14, 15)]. The lectin domain binds to D-mannose (D-Man), and N-acetyl-D-mannosamine (D-ManNAc), N-acetyl-D-glucosamine (D-GlcNAc) and L-fucose (L-Fuc), leading to recognition of numerous pathogens via interaction with pathogen-associated molecular patterns (PAMP) decorating their cells (more details are given in **Tables 2**, **3**). Furthermore, MBL contributes to the elimination of senescent fibroblasts, apoptotic, necrotic and certain cancerous cells (7, 16).

**Table 1:** Genes encoding for human collectins and ficolins and corresponding proteins.

Gene	chromosome	Number of exons	Protein	Number of AA in single (mature) chain	Number of Gly-X-Y repeats	Molecular mass of mature chain (kDa)
*MBL2*	10 (10q11.2-q21)	4	mannose-binding lectin (mannan-binding lectin) MBL	228	19*	32
*COLEC10*	8 (8q23–q24.1)	6	collectin-10 (collectin liver 1) CL-10/CL-L1	246	24	34
*COLEC11*	2 (2p25.3)	7	collectin-11 (collectin kidney 1) CL-11/CL-K1	250	24	34
*COLEC12*	18 (18p11.32)	10	collectin-12 (collectin placenta 1) CL-12/CL-P1	742	49	140
*SFTPA1*	10 (10q22.2-q23.1)	8	surfactant protein A1 SP-A1	248	23*	35
*SFTPA2*	10 (10q22.2-q23.1)	8	surfactant protein A2 SP-A2	248	23*	35
*SFTPD*	10 (10q22-q23)	8	surfactant protein D SP-D	355	59	43
*FCN1*	9 (9q34.3)	9	ficolin-1 (M-ficolin)	297	21*	35
*FCN2*	9 (9q34.3)	8	ficolin-2 (L-ficolin)	288	17*	35
*FCN3*	1 (1p36.11)	8	ficolin-3 (H-ficolin)	276	11	35

*with an interruption inside (see details within the text).

**Table 2:** Pathogens recognised by collectins and ficolins.

Protein	Recognised pathogens
mannose-binding lectin (MBL)	***Bacteria:*** *Actinomyces israelii* *Bacteroides ureolyticus* *Bifidobacterium bifidum* *Brucella abortus* *Brucella melitensis* *Burkholderia cepacia* *Chlamydia pneumoniae* *Chlamydia psittaci* *Chlamydia trachomatis* *Escherichia coli* *Fusobacterium necrogenes* *Fusobacterium nucleatum* *Haemophilus influenzae* *Helicobacter pylori* *Klebsiella aerogenes* *Klebsiella pneumoniae* *Legionella pneumophila* *Listeria monocytogenes* *Micrococcus lysodeictcus* *Mycobacterium avium* complex *Mycobacterium bovis* *Mycobacterium gordonae* *Mycobacterium leprae* *Mycobacterium tuberculosis* *Mycoplasma pneumoniae* *Neisseria gonorrhoeae* *Neisseria meningitidis* *Nocardia farcinica* *Propionibacterium acnes* *Proteus mirabilis* *Proteus vulgaris* *Pseudomonas aeruginosa* *Salmonella enterica* *Staphylococcus aureus* (uncapsulated) *Streptococcus agalactiae* *Streptococcus pneumoniae* *Ureaplasma urealyticum* *Yersinia enterocolitica* ***Fungi:*** *Aspergillus fumigatus* *Candida albicans* *Cryptococcus neoformans* *Saccharomyces cerevisiae* ***Viruses:*** Hepatitis B Herpes simplex 2 HIV Influenza A SARS-CoV-1 SARS-CoV-2 ***Protozoan/worm parasites:*** *Cryptosporidium parvum* *Leishmania mexicana* *Pneumocystis carinii* *Plasmodium falciparum* *Schistosoma haematobium* *Schistosoma mansonii* *Trichinella spiralis* *Trypanosoma cruzi*
collectin-10/-11/their complex (CL-LK)	***Bacteria:*** *Escherichia coli* *Klebsiella pneumoniae* *Mycobacterium tuberculosis* *Pseudomonas aeruginosa* *Streptococcus pneumoniae* ***Fungi:*** *Candida albicans* *Saccharomyces cerevisiae* ***Viruses:*** HIV Influenza A ***Protozoan/worm parasites:*** *Schistosoma haematobium*
collectin-12 (CL-12)	***Bacteria:*** *Escherichia coli* *Staphylococcus aureus* ***Fungi:*** *Aspergillus fumigatus* *Saccharomyces cerevisiae*
surfactant protein A (SP-A)	***Bacteria:*** *Bacillus subtilis* *Chlamydia pneumoniae* *Chlamydia trachomatis* *Escherichia coli* *Haemophilus influenzae* *Klebsiella pneumoniae* *Mycobacterium avium* complex *Mycobacterium bovis* *Mycobacterium tuberculosis* *Mycoplasma pneumoniae* *Mycoplasma pulmonis* *Pseudomonas aeruginosa* *Staphylococcus aureus* *Streptococcus pneumoniae* Group A and B streptococci ***Viruses:*** Herpes simplex Influenza A Respiratory syncytial ***Fungi:*** *Aspergillus fumigatus* *Cryptococcus neoformans* *Pneumocystis jiroveci*
surfactant protein D (SP-D)	***Bacteria:*** *Bacillus subtilis* *Chlamydia pneumoniae* *Chlamydia trachomatis* *Escherichia coli* *Haemophilus influenzae* *Helicobacter pylori* *Klebsiella pneumoniae* *Mycobacterium avium* complex *Mycobacterium tuberculosis* *Mycoplasma pneumoniae* *Pseudomonas aeruginosa* *Staphylococcus aureus* *Streptococcus pneumoniae* ***Viruses:*** HIV Influenza A Respiratory syncytial SARS-CoV-1 SARS-CoV-2 ***Fungi:*** *Aspergillus fumigatus* *Candida albicans* *Cryptococcus neoformans* *Pneumocystis jiroveci* *Saccharomyces cerevisiae*
ficolin-1	***Bacteria:*** *Escherichia coli* *Mycobacterium bovis* *Mycobacterium tuberculosis* *Pseudomonas aeruginosa* *Salmonella enterica* *Staphylococcus aureus* *Streptococcus agalactiae* *Streptococcus mitis* *Streptococcus pneumoniae* ***Viruses:*** Ebola
ficolin-2	***Bacteria:*** *Escherichia coli* *Mycobacterium bovis* *Mycobacterium tuberculosis* *Pseudomonas aeruginosa* *Salmonella enterica* *Staphylococcus aureus* *Streptococcus agalactiae* *Streptococcus pneumoniae* ***Viruses:*** Hepatitis C Influenza A ***Fungi:*** *Aspergillus fumigatus* ***Protozoan/worm parasites:*** *Giardia intestinalis* *Trypanosoma cruzi*
ficolin-3	***Bacteria:*** *Aerococcus viridans* *Escherichia coli* *Hafnia alvei* *Mycobacterium bovis* *Mycobacterium kansasii* *Mycobacterium tuberculosis* *Pasteurella pneumotropica* *Salmonella enterica* ***Viruses:*** Influenza A ***Fungi:*** *Aspergillus fumigatus* ***Protozoan/worm parasites:*** *Giardia intestinalis* *Trypanosoma cruzi*

**Table 3:** Examples of major microbial ligands recognised by collectins and ficolins.

Protein	Examples of recognised pathogen-associated molecular patterns
mannose-binding lectin (MBL)	capsular polysaccharides (CPS), lipopolysaccharides (LPS), mannosylated lipoarabinomannans (ManLAM), mycobacterial antigen 85, fungal mannans, SARS-CoV-1/2 S-glycoproteins (S-gp)
collectin-10/-11 and their complex (CL-LK)	lipopolysaccharides (LPS), mannosylated lipoarabinomannans (ManLAM), fungal mannans, sialyl Lewis^x^, Lewis^Y^ antigens
collectin-12 (CL-12)	zymosan, Lewis^a^, Lewis^x^ antigens
surfactant protein A (SP-A)	capsular polysaccharides (CPS), lipopolysaccharides (LPS), outer membrane proteins; Eap (staphylococcal adhesin); fungal and viral glycoproteins
surfactant protein D (SP-D)	lipopolysaccharides (LPS), lipoarabinomannan (LAM), peptidoglycans (PG), lipoteichoic acids (LTA), fungal β-glucans; viral glycoproteins
ficolin-1	capsular polysaccharides (CPS), mycobacterial antigen 85, viral glycoproteins, sialic acid
ficolin-2	capsular polysaccharides (CPS), lipopolysaccharides (LPS), peptidoglycans (PG), lipoteichoic acids (LTA), β-1,3-glucans, mycobacterial antigen 85, viral glycoproteins
ficolin-3	exopolysaccharides (EPS), lipopolysaccharides (LPS), mycobacterial antigen 85,

The chains of CL-10 and CL-11 (34 kDa) consist of 246 and 250 AA residues, respectively (**Table 1**; **Figure 2A**). Their N-terminal domains differ in length (15 and 19 AA, respectively) whilst the collagen-like, neck and carbohydrate recognition domains consist of an identical number of residues: 72 (24 Gly-X-Y repeats), 34, and 125, respectively, but with distinct sequences [reviewed in (17–19)]. The basic subunits may be homo- or heterotrimers (consisting of one CL-10 and two CL-11 polypeptides). In the latter case, the molecule is called CL-LK (20). Those trimers undergo further oligomerization (up to octadecamers) (17, 20). The ligand specificity of CL-LK is rather broad, and similar to some extent to that of MBL (21, 22). That probably reflects a synergistic effect between CRDs of CL-10 and CL-11. CL-10 recognises D-Man, L- and D-fucose, D-galactose (D-Gal), and D-GlcNAc (19, 23) while CL-11 recognises the D-Man-(α1,2)-D-Man disaccharide (24) as well as D-Man, L-Fuc and D-ManAc (19, 25). That enables CL-LK to bind to a variety of pathogens as well as apoptotic cells [reviewed in (19, 22)]. Furthermore, Kirketerp-Moller et al. (26) reported specific recruitment of CL-11 to ligands by C1q/TNF-related protein (CTRP) and subsequent complement activation. Details concerning those microbes and associated molecular patterns are listed in **Tables 2**, **3**.

Collectin-12 was initially described as a scavenger transmembrane receptor, expressed in vascular endothelial cells and in placenta (27, 28). It was later found on other cells (including macrophages, astrocytes and microglia) as well as in a soluble form (8, 29). Its ectodomain includes carbohydrate recognition (126 AA), neck (17 AA) and collagen-like (147 AA) regions preceded by a long (223 AA) coiled-coil fragment (**Table 1**; **Figure 2A**). Due to that and additional transmembrane and intracellular domains, the MW of its single chain is higher than that of other members of the subfamily (140 kDa, 742 amino acid residues) Furthermore, it exists as lower oligomers (one to three subunits) (8, 26, 27). Collectin-12 recognises D-GalNAc, D-Gal, Lewis^x^ (Le^x^), T-antigen and Tn-antigen. The Le^x^ is the D-Gal-(β1,4)-L(Fuc-(α1,3)-D-GlcNAc trisaccharide motif, present in some self-cells but also in surface structures of such pathogens as *Helicobacter pylori, Streptococcus sanguinis* or *Schistosoma mansoni* eggs. It should however be stressed that CL-12 does not bind to its sialylated (commonly expressed) form (sLe^x^). Tumour-associated T- and Tn-antigens contain both carbohydrate and amino acid residues: D-Gal(β1,3)-D-GalNAc-Ser/Thr and D-GalNAc-Ser/Thr, respectively (18, 30–34). For more details see **Tables 2**, **3**.

Human surfactant protein A exists in two forms, SP-A1 and SP-A2, closely related but synthesized under the control of distinct genes (see below). The SP-A subunits may be homo- or heterotrimers. The chains of both forms are built up of 248 amino acid residues (35 kDa), with 27 in cysteine-rich, 73 in collagen-like, 33 in neck and 115 in carbohydrate recognition domains. The collagen-like region includes 23 Gly-X-Y repeats and, like MBL, an interrupting sequence (Pro-Cys-Pro-Pro, after the 13^th^ triplet) (**Table 1**; **Figure 2A**), resulting in a “tulip bouquet” shape of the multimer (up to 6 subunits). SP-A has affinity for D-ManNAc, D-Man, L-Fuc, D-maltose (D-Mal), D-glucose (D-Glc) and D-GlcNAc (13, 35–40). It moreover recognises non-carbohydrate bacterial ligands, as extracellular adherence protein (Eap adhesin), a virulence factor of *Staphylococcus aureus* (41). Pathogens and corresponding molecular patterns recognised by SP-A are presented in **Tables 2**, **3**.

The polypeptide chain of another surfactant protein, SP-D (355 amino acid residues, 43 kDa) is characterised by a long collagen-like region (59 Gly-X-Y repeats, no kink). The cysteine-rich region, coiled-coil segment and CRD are built-up from 24, 46 and 106 AA, respectively (**Table 1**; **Figure 2A**). Basic subunits oligomerise to form tetramers which may further multimerise (up to 8 tetramers/96 chains). The lectin domain of SP-D binds to D-Mal, D-Man, L-Fuc, D-GlcNAc, D-Glc and D-Gal. It furthermore is able to recognise inositol and a disaccharide, lactose (13, 37–40, 42, 43). Microbes and their basic structures that are ligands for this collectin are listed in **Tables 2**, **3**.

#### 1.1.2 Genes encoding collectins and their polymorphisms

Genes encoding MBL (*MBL2*), SP-A (*SFTPA1, SFTPA2*) and SP-D (*SFTPD*) are localised to chromosome 10 (10q21-24) within a cluster including also MBL- and SP-A-related pseudogenes. Genes encoding MBL and SP-A contain four exons while that for SP-D has eight (**Table 1**). All of them are highly polymorphic. The majority of disease-association studies of the *MBL2* gene has been focused on 6 (or less) polymorphisms: [rs11003125 (-550 G>C, commonly termed H/L), rs76206 (-221 G>C known as Y/X)] (both localised to the promoter region), [rs7095891 (+4 C>T, P/Q] (at 5’untranslated region), rs5030737 (+223 C>T, R52C, commonly called A/D), rs1800450 (+230 G>A, G54D, A/B) and 1800451 (+239 G>A, G57E, A/C)], all from exon 1. Variant (minority) alleles D, B, and C are collectively named O [reviewed in (15)]. Due to strong linkage disequilibria, only seven haplotypes are considered common (HYPA, LYPA, LYQA, LXPA, LYPB, LYQC, HYPD), differently distributed among various ethnic groups. All those polymorphisms affect the concentration of MBL in blood: those in promoter via influencing the level of gene expression, those in exon 1 via influencing protein half-life (shortened for products related to O variants due to higher sensitivity to serum metalloproteases). Additionally, presence of D, B and/or C allele leads to lower MBL activity. The O/O and LXPA/O (or LXA/O, XA/O) genotypes are associated with MBL primary deficiency (considered the commonest human immunodeficiency) but in some individuals carrying other genotypes (including not only A/O but also A/A), this lectin may be undetectable or present in serum at a very low level. Generally, huge inter-genotype differences are not uncommon (15). Moreover, certain polymorphisms of the 5’UTR of exon 4 have been described which are believed to be clinically relevant, mainly in relation to cancer, therefore they will not be discussed here.

The genes encoding SP-A1 (*SFTPA1*) and SP-A2 (*SFTPA2*) are in linkage disequilibrium and are located in opposite transcriptional orientation. Combinations of their polymorphisms localised to the coding regions were used for establishing several frequent variants called 6A, 6A^2^, 6A^3^, 6A^4^, 6A^5^ (for *SFTPA1*) or 1A, 1A^0^, 1A^1^, 1A^2^, 1A^3^, 1A^5^ (for *SFTPA2*), affecting protein activity [reviewed in (40, 44, 45)]. Details are given in **Table 4**. Among 14 included polymorphisms, 8 are silent, associated with single nucleotide but not amino acid residue exchanges; 5 of them differentiate between *SFTPA1* and *SFTPA2* genes. Furthermore, both gene and AA sequences differ at 4 additional positions: codons 66 (rs1059049; ATG/Met for SP-A1 and ACA/Thr for SP-A2), 73 (rs1059052; GAT/Asp and AAT/Asn, respectively), 81 (rs1059053; ATC/Ile and GTC/Val, respectively), and 85 (rs1059053; TGT/Cys and CGT/Arg, respectively), all localised to the collagen-like domain. Interestingly, as SNP in codons 9 and 19 are related to the signal peptide-encoding sequence, there are several pairs of *SFTPA1* or *SFTPA2* variants giving identical mature proteins (6A and 6A^3^, 1A and 1A^5^, 1A^0^ and 1A^2^, 1A^1^ and 1A^3^) (40) (see **Table 4**). The most common variants are 6A^2^and 1A^0^. Additionally, some splicing (called A, B, B’, C, C’, D, D’) or sequence variations of both genes at their 5’ and 3’UTR, respectively, were reported to affect their expression (44, 45).

Table 4Common polymorphisms localised to the coding region of the *SFTPA1* (SP-A1) (A) and *SFTPA2* (B) genes, and corresponding variants (related codons and amino acid residues are given).

**Table:** 

A						
SNP	codon	Nucleotide triplets/amino acids				
		6A	6A^2^	6A^3^	6A^4^	6A^5^
rs139899873	9	AAC/Asn	AAC/Asn	AAC/Asn	AAC/Asn	AAC/Asn
rs1059047	19	GCG/Ala	GTG/Val	GTG/Val	GTG/Val	GCG/Ala
rs1136450	50	CTC/Leu	GTC/Val	CTC/Leu	CTC/Leu	CTC/Leu
rs1136451	62	CCG/Pro	CCA/Pro	CCA/Pro	CCG/Pro	CCG/Pro
rs1059051	71	GGA/Gly	GGA/Gly	GGA/Gly	GGA/Gly	GGA/Gly
rs1136452	91	CCT/Pro	CCT/Pro	CCT/Pro	CCT/Pro	CCT/Pro
rs1136452	94	AGG/Arg	AGG/Arg	AGG/Arg	AGG/Arg	AGG/Arg
rs1059056	114	TTT/Phe	TTT/Phe	TTT/Phe	TTT/Phe	TTT/Phe
rs1059057	133	ACG/Thr	ACA/Thr	ACA/Thr	ACA/Thr	ACA/Thr
rs3997777	140	TCC/Ser	TCC/Ser	TCC/Ser	TCC/Ser	TCC/Ser
rs1059058	202	GAC/Asp	GAC/Asp	GAC/Asp	GAC/Asp	GAC/Asp
rs876657998	216	CCC/Pro	CCC/Pro	CCC/Pro	CCC/Pro	CCC/Pro
rs4253527	219	CGG/Arg	CGG/Arg	CGG/Arg	TGG/Trp	TGG/Trp
rs397728201	223	CAG/Gln	CAG/Gln	CAG/Gln	CAG/Gln	CAG/Gln

**Table:** 

B							
SNP	codon	Nucleotide triplets/amino acids					
		1A	1A^0^	1A^1^	1A^2^	1A^3^	1A^5^
rs1059046	9	ACC/Thr	AAC/Asn	ACC/Thr	ACC/Thr	AAC/Asn	ACC/Thr
rs201847938	19	GCG/Ala	GCG/Ala	GCG/Ala	GCG/Ala	GCG/Ala	GCG/Ala
rs192907309	50	GTC/Val	GTC/Val	GTC/Val	GTC/Val	GTC/Val	GTC/Val
rs2434114	62	CCG/Pro	CCG/Pro	CCG/Pro	CCG/Pro	CCG/Pro	CCG/Pro
rs143780551	71	GGG/Gly	GGG/Gly	GGG/Gly	GGG/Gly	GGG/Gly	GGG/Gly
rs17886395	91	CCT/Pro	GCT/Aln	GCT/Aln	GCT/Aln	GCT/Aln	CCT/Pro
rs17886221	94	AGA/Arg	AGA/Arg	AGA/Arg	AGA/Arg	AGA/Arg	AGA/Arg
rs147679203	114	TTC/Phe	TTC/Phe	TTC/Phe	TTC/Phe	TTC/Phe	TTC/Phe
rs763971475	133	ACA/Thr	ACA/Thr	ACA/Thr	ACA/Thr	ACA/Thr	ACA/Thr
rs1965707	140	TCC/Ser	TCC/Ser	TCT/Ser	TCC/Ser	TCT/Ser	TCT/Ser
rs17880902	202	GAT/Asp	GAT/Asp	GAT/Asp	GAT/Asp	GAT/Asp	GAT/Asp
rs17096771	216	CCT/Pro	CCT/Pro	CCT/Pro	CCT/Pro	CCT/Pro	CCT/Pro
rs571610539	219	CGG/Arg	CGG/Arg	CGG/Arg	CGG/Arg	CGG/Arg	CGG/Arg
rs1965708	223	CAG/Gln	CAG/Gln	AAG/Lys	CAG/Gln	AAG/Lys	CAG/Gln

The *SFTPD* (SP-D) gene is located in the same transcriptional orientation as *SFTPA2.* It contains 8 exons (7 coding) (**Table 1**). Its polymorphisms, located in 3’UTR, protein encoding region and 5’UTR are known to influence SP-D concentration/activity and/or to have a variety of disease associations (44, 46, 47). Some of those located within the protein encoding exons are silent [rs6413520 (+75 T>C, Ser25Ser), rs1051246 (+858 T>C, Ala286Ala)], others are related to amino acid substitutions [rs721917 (+32 T>C, Met11Thr), rs2243639 (+478 A>G, Thr160Ala), rs3088308 (+868 T>A, Ser270Thr)] (44).

The human *COLEC10* gene (encoding for CL-10/CL-L1) is localised to chromosome 8 and contains 6 exons (**Table 1**). It is highly conserved (as is *COLEC11*, for CL-11/CL-K1) and the majority of its polymorphisms is located in non-coding regions (48). However, two *COLEC10* polymorphisms are associated with amino acid exchange: rs150828850 (+23881 A>C, Glu78Asp, affecting structure of the collagen-like region) and rs149331285 (+36545 C>T, Arg125Trp, affecting structure of the neck region and CL-10 concentration in serum) (17, 18, 22, 48).

The *COLEC11* gene (chromosome 2; 7 exons) (**Table 1**), in contrast to *COLEC10*, produces a variety of transcript isoforms. However, two only were confirmed to be translated and detected in circulation: a or f (full-length, 34 kDa chain) and d (lacking part of the collagen-like region encoded by exon 8; 31 kDa) (18, 20, 21). Like in the case of *COLEC10*, most of *COLEC11* SNP is located within promoter or introns. One of them, rs3820897 (-9570 C>T, promoter region) was reported to influence serum level of CL-11. The non-synonymous polymorphism in exon 7, rs7567833 (+39618 C>G, His219Arg) is considered to affect ligand binding capacity but not concentration in blood (17, 22, 48).

The gene encoding collectin-12, *COLEC12*, is located on chromosome 18 and contains 10 exons (**Table 1**). Two major isoforms (I – full length and II – lacking CRD) and several others were found to be transcribed (17, 27, 33). Ohmori et al. (49) identified six polymorphisms, including three related to coding exons: one silent, in exon 5 (+800 C>T, Thr267Thr) and two non-synonymous, in exon 6 (+1563 C>T, Ser522Pro and +1815 A>G, Gly606Ser) and constructed 22 related haplotypes (49). However, the GeneCards database mentions several other SNP which influence amino acid sequence: +182 T>C (Val61Ala), +319 G>A (Glu 107Lys), +494 C>G (Thr165Ser), +707 G>A (Arg236Gln) (https://www.genecards.org/cgi-bin/carddisp.pl?gene=COLEC12).

#### 1.1.3 Structure and specificity of ficolins

Unlike collectins, ficolins have a globular, fibrinogen-like domain (FBG) at their C-termini. Therefore, the name of this protein family comes from FIbrinogenCOllagenLectIN, although the FBG region is not a typical lectin domain. Three human ficolins have been described: ficolin-1 (or M-ficolin), ficolin-2 (or L-ficolin) and ficolin-3 (or H-ficolin), encoded by the *FCN1, FCN2* and *FCN3* genes, respectively. All of them, by forming complexes with MASP, are able to activate complement via the lectin pathway [reviewed in (50, 51)]. Importantly, ficolin-2 was demonstrated to interact with CRP and PTX3 leading to enhancement of recognition of some pathogens and boosting complement-mediated killing via lectin/classical pathways (52, 53). Similar cross-talks of ficolin-1 are associated with amplification of complement activation and a modulatory effect on secretion of inflammatory cytokines (54, 55) (**Figure 1**).

The mature polypeptide chains of ficolins consist of 297 (ficolin-1), 288 (ficolin-2) or 276 (ficolin-3) AA residues (molecular weight 34 - 35 kDa), including 21, 17 or 11 Gly-X-Y repeats in their collagen-like regions, respectively. After the second repeat in ficolin-1 and ficolin-2 there is a six amino acid interruption. The ficolin-1, ficolin-2 and ficolin-3 N-terminal regions encompass 10, 13 and 24 AA, neck domains of 9, 9 and 12 AA, respectively. The C-terminal FBG region has 209, 209 and 207 AA, respectively, in ficolins 1 to 3. (**Table 1**; **Figure 2B**). Single chains generally form homotrimeric basic subunits via disulphide bonds, due to the presence of cysteine residues in their N-terminal domains, which further oligomerize, usually into dodecamers or octadecamers (50, 56, 57). Moreover, it was evidenced that ficolin-2 and ficolin-3 may form heterocomplexes (called ficolin-23), supposed to include homotrimers of both or heterotrimers (58).

Ficolins generally exhibit affinity to acetylated sugars but also to some non-acetylated structures. Namely, ficolin-1 binds D-GlcNAc, N-acetyl-D-galactosamine (D-GalNAc), D-ManNAc and sialic acid; ficolin-2 binds D-GlcNAc, D-GalNAc, D-ManNAc, N-acetyl-neuraminic acid (NeuNAc), N-acetylated cysteine, acetylcholine and heparin; and ficolin-3 binds D-GlcNAc, D-GalNAc, D-Gal and D-Fuc [reviewed in (5, 50, 51)]. Unlike other proteins of this family, ficolin-2 has four distinct binding sites that give it a broad specificity (59–62), which is further extended by cross-talk with short and long pentraxins. Details concerning microbial ligands for human ficolins are presented in **Tables 2**, **3**.

#### 1.1.4 Genes encoding ficolins and their polymorphisms

The *FCN1* (9 exons) and *FCN2* (8 exons) genes are located on chromosome 9q34 (**Table 1**). Due to their localisation, exon organisation and high sequence homology (approx. 80% at the amino acid level) it is assumed that they originate from gene duplication (63, 64). The *FCN3* gene, containing 8 exons, is located on chromosome 1p36.11) (**Table 1**). It was suggested to branch out by gene duplication in the early stage of evolution of *Vertebrata.* The amino acid sequence of its product, ficolin-3, shows approx. 48% homology with both other ficolins (63, 64).

All three genes are highly polymorphic and distribution of their variants differs in various populations/ethnic groups [discussed in details in (63, 64)]. Some of them were demonstrated to influence expression level or activity of ficolins, and to have clinical associations. Ammitzboll et al. (65) found minor alleles for three promoter [rs7857015 (-1524 T>C), rs10120023 (-542 G>A), rs10117466 (-144 C>A)] and one exon 1 [rs10858293 (+33 G>T, Gly11Gly) SNP of the *FCN1* gene (all in strong linkage disequilibrium) to be associated with high concentrations of ficolin-1 in serum. Earlier, Munthe-Fog et al. (66) reported such a relationship for rs10120023 and rs10117466 (ficolin-1 levels were determined in plasma) and found moreover lower expression of *FCN1* mRNA in granulocytes and monocytes in carriers of G and/or C alleles, respectively. Regarding non-synonymous polymorphisms, variant alleles at rs148649884 [+6658 G>A, Ala218Thr (exon 8)] and rs138055828 [+7959 A>G, Asn289Ser (exon 9)] were found associated with lower serum concentrations of ficolin-1 while those at rs147309328 [+4759 G>A, Arg124Gln (exon 6)] and rs56084543 [+4837 C>T, Thr150Met (exon 6)] had the opposite effect. Furthermore, the presence of threonine at position 218 and serine at 289 affected ligand binding capacity. Another SNP, localised to exon 9, rs150625869 (+7895 T>C, Ser268Pro) was deduced to cause total ficolin-1 deficiency in a homozygous state, however no such case has been identified to date (65).

Similarly, no primary (genetically-determined) instance of ficolin-2 deficiency has been found. Minor alleles corresponding to three promoter region SNP of the *FCN2* gene were associated with low ficolin-2 concentration in blood: rs3124952 (-986 A>G), rs3811140 (-557 A>G) and rs7865453 (-64 A>C) whereas minor alleles at rs3124953 (-602 G>A) and rs17514136 (-4 A>G) were associated with higher levels (67–70). Moreover, variant alleles for rs17549193 (+6359 C>T, Thr236Met) and rs7851696 (+6424 G>T, Ala258Ser) (both located in exon 8) are related to higher and lower concentrations of the gene product, respectively, and also affect affinity to the ligand (D-GlcNAc) (67–70). Due to linkage disequilibria, their effect on ficolin-2 concentration is quite ambiguous and marked differences in the level of this protein in serum are observed among carriers of the same genotype, even between monozygotic twins (67, 68, 70). More recently, Świerzko et al. (71) found two *FCN2* gene 3’UTR polymorphisms, rs4521835 (T>G) and rs73664188 (T>C), to influence ficolin-2 concentration significantly: the corresponding GC haplotype was associated with a low level of this protein in the cord serum (71).

Rare ficolin-3 deficiency is associated with a single nucleotide deletion in exon 5 (+1637delC; rs28357092), leading to a frame shift causing an early stop codon and lack of protein synthesis (63, 72). Described originally in heterozygotes, it was hypothesized to cause ficolin-3 deficiency in homozygotes (72). Indeed, Munthe-Fog et al. (73) identified the first case soon after. The patient had severe and recurrent infections (73). Later, several other cases were described, including newborns (discussed below). A dozen or so other *FCN3* polymorphisms, within the promoter region, exons and introns have been described in various populations, mostly with low (<0.1) minor allele frequency (63, 64, 72). Interestingly, promoter SNPs appear to have no significant impact on the concentration of ficolin-3 in serum (72). The most common in European (Danish) population, rs41415450 (-325 G>A; MAF=0.11) was not observed in Africans (Ghana, Mozambique) or Japanese (63, 64).

## 2 Clinical associations of collectins in the neonate

### 2.1 Mannose-binding lectin

The most extensively investigated complement-activating collectin in the context of neonatal health and disease is MBL. Several, differently designed, studies have found significant correlations between cord blood MBL concentration and gestational age (74–79). Lau et al. (74) found that preterms having low MBL concentration in serum (defined as <400 ng/ml) were born at shorter gestational age than babies with higher levels of this collectin. They moreover observed generally lower MBL in preterm compared with term babies what was further confirmed by Hilgendorff et al. (76) and Sallenbach et al. (79). Van der Zwet et al. (78) reported higher frequency of low MBL-associated haplotypes in preterm compared with term neonates. However, Kilpatrick et al. (80) found no correlation with gestational age. Moreover, data from a large (n>1800) cohort of consecutively recruited newborns revealed neither significant correlation of MBL with GA nor a greater difference in median MBL concentration in cord sera between term and preterm newborns. No association of *MBL2* genotypes or MBL concentrations in cord sera with birthweight was observed (81). These conflicting findings are difficult to reconcile. Complicating matters further, low MBL-dependent lectin pathway activity was found to be associated with prematurity (81).

Some contradictory data were also published in the context of associations of *MBL2* genotypes in general and/or genetically-determined MBL deficiency with spontaneous preterm births. Bodamer et al. (82) reported the D allele (corresponding to codon 52 polymorphism, rs5030737) to be a risk factor and L/L homozygosity (related to promoter SNP at position -550, rs11003125) to have the opposite effect. Furthermore, O/O genotypes tended to be more common in preterm compared with term babies (82). In contrast, Swierzko et al. (81) found A/A *MBL2* genotypes [where A corresponds to major alleles in codons 52 (A/D, rs5030737), 54 (A/B, rs1800450) and 57 (A/C, rs1800451)] to be significantly more frequent among preterms. That finding was later partially confirmed by Carvalho da Silva et al. (83) who genotyped neonates and their mothers. They found maternal/neonatal LYA haplotypes to be associated with preterm birth and the LYB haplotype to be protective. Chan et al. (84) related spontaneous preterm birth with depletion of *Lactobacillus* sp. from the vaginal microbiome leading to a maternal inflammatory response expressed through increased concentration of MBL, C3b, C5, IL-6, IL-8, IL-1β, IgM and IgG. Therefore, they suggested cross-talk between the innate and adaptive defence mechanisms, bridged by complement activation, to enhance the risk of preterm delivery. Earlier, Frakking et al. (77), Karjalainen et al. (85) and Grumach et al. (86) found no difference of frequency of *MBL2* genotypes between preterm and term neonates. Moreover no impact of maternal genotype was reported by Karjalainen et al. (85).

One of the leading causes of prematurity is preterm premature rupture of membranes (pPROM). Modi et al. (87, 88), using whole genome sequencing, identified the rs74754826 mutation of the *MBL2* gene [codon 210, + 628 G>T, Glu210Ter (introducing “stop” codon)] as a risk factor for pPROM in African Americans. This mutation introduces a stop codon, resulting in deletion of 38 terminal amino acid residues in the CRD.

Premature births are often associated with very low birthweight (VLBW). The earliest reports suggested *MBL2* promoter region polymorphisms associated with low circulating MBL were linked to low body mass (89). However, Gibson et al. (90) observed a high prevalence of the LYPA haplotype (corresponding to relatively high MBL concentration in serum/activity) in small for gestational age, (SGA, birthweight <10 percentile) premature (born at GA <32 weeks) babies. In contrast, Cakmak et al. (91) reported the codon 54 variant (B) allele (commonly related to LYPB haplotype) and low MBL concentration to be associated with low birthweight in a corresponding gestational age group while Hartz et al. (92) reported no impact of MBL on incidence of VLBW.

There is more agreement, but by no means total consistency, in the extensive literature concerning MBL and neonatal sepsis. Frakking et al. (93) observed associations of low MBL and its genetically-determined deficiency not only with early onset sepsis, but also with culture-proven sepsis and pneumonia; in that study, both preterm and term newborns were recruited. Several other groups found lower serum MBL levels in cases of neonatal sepsis compared with controls as well (90, 94–99). Dzwonek et al. (100) based on data from preterms (GA 24-36 weeks) demonstrated that *MBL2* A/O and O/O genotypes are risk factors for sepsis. Koroglu et al. (101) found codon 54 (A/B) and 57 (A/C) SNP to be associated with a higher risk of sepsis (especially early onset) but not culture-proven sepsis, while Ozdemir et al. (102) noted lower MBL levels related with sepsis in general and especially with culture-proven sepsis in neonates born at GA ≤34 weeks with fetal inflammatory response syndrome. Similarly, Wahab Mohamed and Saeed (103) published data from both term and preterm babies demonstrating significantly lower MBL levels in individuals with confirmed sepsis (and especially septic shock) than in the control (infection-free) group. Świerzko et al. (104) observed significantly lower median MBL concentrations in babies with diagnosed sepsis compared with the control group (no infection till hospital discharge). However, no significant differences were found between newborns who had a milder disease course (infection but not sepsis) and controls or between disease groups (104). Hartz et al. (105), investigating a large cohort of very low birthweight infants (VLBWI) found that total MBL deficiency (undetectable in serum) is related to sepsis in babies born at GA ≥32 ≤ 37 weeks only. Dogan et al. (106) suggested MBL concentrations <700 ng/ml enhanced the risk of late-onset sepsis in preterms. Both Dogan et al. (106) and Schlapbach et al. (107) found the strongest association of MBL deficiency to be with Gram-negative sepsis. Recently, Elhawary et al. (99) reported significant association of B allele with sepsis in preterms.

The association with deficiency of circulating MBL could be misleading, as the relationship is not as readily apparent at the DNA level. Auriti et al. (95) found no significant difference in the frequencies of *MBL2* genotypes between infected and noninfected neonates, despite a marked difference in serum MBL concentrations. Their findings are supported by several other studies reporting no association between neonatal sepsis and either *MBL2* genotype or the presence of the B allele (78, 97, 108–110). These findings are, of course, in contrast to some of the reports cited earlier, so collectively are hard to reconcile.

Meta-analyses of available data have concluded that the codon 54 SNP variant influences risk for development of neonatal sepsis in general (111) or culture-proven sepsis (112, 113). However MBL concentration was estimated to be of only moderate prognostic value (111).

The relationship of MBL to neonatal bronchopulmonary dysplasia (BPD) is also unclear. Hilgendorff et al. (109) reported that X and B *MBL2* variants are risk factors for BPD. A harmful effect of B/B and protective influence of A/A genotype, respectively, were later postulated by Cakmak et al. (91). Furthermore, Capoulongo et al. (114) found the A/D genotype to be a BPD risk factor and the L allele to be beneficial. Later, Speletas et al. (115) reported a significant association of XA/O and O/O genotypes with transient tachypnea and respiratory distress syndrome (RDS) (their cohort comprised of both term and preterm neonates). On the other hand, Koroglu et al. (101) found no relationship between *MBL2* genotype and BPD or RDS.

### 2.2 Collectin-10, collectin-11 and collectin-12

Both collectin-10 and collectin-11 are considered to be essential for fetal development: a variety of mutations of *COLEC10, COLEC11* and *MASP1/3* genes (the last one encodes MBL-associated serine proteases-1, -3 and non-enzymatic MAp-44, forming complexes with MBL, CL-10, CL-11 and ficolins) were reported in patients diagnosed with Malpuech, Michels, Mingarelli and Carnevale (3MC) syndrome, with craniofacial, renal or genital abnormalities, growth and intellectual disability [reviewed in (22)].

Although collectin-12 (or collectin placenta-1) was demonstrated to recognise certain pathogens, including *Aspergillus fumigatus, Staphylococcus aureus* and *Escherichia coli* (34, 116), to activate complement via both alternative and classical pathways (8–11) and its soluble form was detected in cord plasma (in contrast to plasma from adult donors) (8), there is no data published concerning its significance in neonatal infections. On the other hand, like CL-10 and CL-11, it is considered crucial for ontogenesis. McLennan et al. (117), based on computational analysis and data from an animal model (chick embryo), found CL-12 contributed to cranial neural crest cell trajectories and promoted collective cell migration. Recently, Ajami et al. (118) described siblings (3.5 year-old girl and 10 month-old boy) with derivative chromosome 9, resulting in partial 9p monosomy and 18p trisomy. Fifty-three and twenty-two (including *COLEC12*) affected genes were identified for the deletion and duplication, respectively. Both patients were born with dysmorphic features and congenital malformations. At the time of examination, besides craniofacial abnormalities, some intellectual/developmental disabilities were also found. Furthermore, a cryptorchidism and incorrect brain magnetic resonance image (demonstrating delayed myelination, enlarged sylvian cistern, cavum septum pellucidum, hypoplastic corpus callosum and trigonocephaly) was documented in the male sibling (118). Although *COLEC12* was one of several translocated genes, the clinical manifestation reported may confirm its importance for embryo-/ontogenesis.

### 2.3 Surfactant protein A and surfactant protein D – collectins which do not initiate complement activation

The complement non-activating collectins, surfactant proteins A and D (SP-A, SP-D) are present in the female reproductive system and are considered to protect from intrauterine infections (which are risk factors for miscarriages, premature births and neonatal sepsis) [reviewed in (13, 119)]. During pregnancy, they are detectable in chorion, amnion, amniotic fluid and placenta (120–125). Snegovskikh et al. (126) postulated that decidual SP-A regulates expression of prostaglandin F2α (PGF2α) in the uterus and therefore contributes to the prevention of premature birth. They moreover excluded association of rs1136451 polymorphism of the *SFTPA1* gene (codon 62, + 186 G>A, Pro62Pro) with prematurity (126). Lower SP-A (and concomitantly higher SP-D and MBL) expression was found in term placentas from spontaneous deliveries compared with caesarean section deliveries (127) which partially confirmed and extended the afore-mentioned data published by Snegovskikh et al. (126). Furthermore, Chaiworapongsa et al. (128) observed lower median SP-A in amniotic fluid samples from women at term in labour compared with those not in labour. Earlier, Pryhuber et al. (120) noticed an increase of amniotic fluid SP-A levels with duration of gestation, especially during third trimester. The *SFTPA1* mRNA expression level in chorioamniotic membranes did not differ between similarly defined groups, but it was higher compared with membranes from preterm deliveries (123). Miyamura et al. (121) reported a marked increase of SP-A (and moderate SP-D) concentration in amniotic fluid from 32^nd^ week of gestation towards term. Interestingly, data from a murine model suggested that SP-A produced by the fetal lung may act as a signal for initiation of delivery [reviewed in (129)]. In contrast, Karjalainen et al. (85) demonstrated that the Met allele (corresponding to *SFTPD* Met31Thr SNP) in babies (but not in their mothers) is associated with spontaneous preterm births and suggested it contributes to a parturition-inducing signal. They however found no similar relationship for *SFTPA1, SFTPA2* or *MBL2* polymorphisms.

SP-A may be detected in terminal bronchioles and alveolar spaces after the 35^th^ week of gestation. Its amount in the alveoli was demonstrated to increase perinatally and to decrease after the 1^st^ week of life (130). The SP-A concentration in umbilical cord serum did not differ depending on gestational age, birthweight, delivery mode or Apgar score (131).

The median concentration of SP-D in umbilical cord sera from term babies was reported to be higher than in preterms born at GA 28-32 weeks but not with those born at shorter GA. Levels of this lectin in tracheal aspirates positively correlated with gestational age and birthweight (76). Similarly, SP-D concentration in bronchoalveolar lavage fluid (BALF) from extremely preterm babies was found to rise with increasing GA (132). In contrast, Dahl et al. (133) found higher SP-D in cord blood from newborns with lower GA [further confirmed by Briana et al. (134)] and no significant correlation with BW. On the other hand, concentrations of this lectin in capillary blood sera (taken postnatally, at age 4-10 days) were decreased with increased body mass. Interestingly, SP-D levels in capillary serum were higher in babies born by caesarean section (CS) than in those born vaginally, whilst cord serum concentrations did not differ significantly. However, rupture of membranes for >1 h was associated with lower SP-D in cord blood within CS group. In neonates delivered vaginally, cord serum SP-D was higher when born within 1 h of labour. Furthermore, significant relationships with maternal age and smoking status (lower concentration in babies from the youngest women and from smokers) were noted (133). Later, analysing data from preterm newborns only, Dahl et al. (135) confirmed relationships of SP-D levels in cord serum with membrane rupture and observed significant association of capillary serum SP-D with that complication, mode of delivery and maternal smoking. Furthermore, concentration of this collectin in cord blood from babies diagnosed with intrauterine growth restriction (IUGR) was lower in comparison with those appropriate for GA (135). In contrast, Briana et al. (134) found an opposite relationship in term newborns. Another discrepancy to the above-mentioned report published by Dahl et al. (133) was higher levels of SP-D in newborns delivered vaginally compared with those born via CS. They furthermore observed an inverse correlation between cord serum SP-D and gestational age (134). Both Dahl et al. (133, 135) and Briana et al. (134) noted SP-D levels in sera taken postnatally were higher than those from cord blood. It has been postulated that concentration of this collectin in cord serum in IUGR patients may be a marker predicting lung function impairment in adulthood (136).

Preterm delivery is often a result of intrauterine infection, which may be manifested by chorioamnionitis. Han et al. (123) found *SFTPA1*-specific mRNA expression to be significantly higher in chorioamniotic membranes from preterm deliveries associated with chorioamnionitis compared with those not. Hilgendorff et al. (76) observed lower SP-D concentrations in sera from preterms born with symptoms of chorioamnionitis. Chaiworapongsa et al. (137) found amniotic fluid concentrations of both SP-A and SP-D to be unchanged. Interestingly, recent data published by De Luca et al. (132) demonstrated higher SP-D levels in BALF from extremely preterm babies affected by chorioamnionitis with fetal involvement compared with the corresponding reference group.

Although no difference in SP-D concentration in cord sera was noted between preterm babies diagnosed with congenital sepsis and without infection (76), the levels of this protein were higher in endotracheal aspirates from preterms aged 1-20 days in comparison with those from controls (aged 1-30 days) (138).

Surfactant deficiency, which is not uncommon among preterms, contributes to a variety of adverse effects, including high susceptibility to respiratory infections and neonatal respiratory distress syndrome (RDS) both of which may in turn lead to bronchopulmonary dysplasia (BPD) or chronic lung disease (CLD). However, those pathologies are not restricted to prematurity-associated impairment of synthesis of surfactant components. Hilgendorff et al. (109) observed elevated concentrations of SP-D in tracheal aspirates from neonates born between 28^th^ and 32^nd^ weeks of gestation but not from extremely preterm babies GA <28 weeks). Later, they found a protective role of minor allele homozygosity for rs1923537 polymorphism of the *SFTPD* gene (+11208 A*>*G, localised to 3’UTR) in newborns <32 weeks of GA. The variant allele in affected individuals was associated with lower prevalence for need for surfactant, oxygen supplementation and for diuretics (139). A relationship between the SNP and RDS in very preterm infants was further confirmed by Ryckman et al. (140). Several other SP-D variants, related to SNP localised to coding region or 5’UTR of the *SFTPD* gene have been considered as RDS risk/outcome modifying factors. Sorensen et al. (141), based on analysis of seven polymorphisms, proposed four of them to be associated with respiratory outcome in preterm babies: rs1923534 (5’UTR), rs721917 (+32 T>C, Met11Thr), rs2243639 (+478 G>A, Ala160Thr; referred also as rs17885900), and rs3088308 (+868 T>A, Ser270Thr). Carrying of the major alleles for rs1923534, rs721917 and rs3088308 was associated with higher risk of respiratory distress, need for oxygen supplementation and respiratory support. On the other hand, three of seventeen reconstructed haplotypes (with frequency >0.05), including minor alleles for rs1923534, rs721917 and rs3088308 were related to lower concentration of SP-D, lower risk of respiratory distress, need for oxygen supplementation and respiratory support (141). In contrast, Chang et al. (142) found no significance of rs721917 or rs2243639. That was later confirmed by Amatya et al. (143), however an intriguing intergenic SNP interactions model was established in their paper, based on investigation of 17 polymorphisms of the *SFTPA1, SFTPA2, SFTPB, SFTPC* and *SFTPD* genes. That led to the identification of a protective effect of rs17886395 (*SFTPA2*) and rs721917 (*SFTPD*) interaction, when both had a dominant effect. Analysis of three-SNP interactions revealed six with *SFTPD* rs721917 or rs2243639 dominant effect involvement. With one exception, combination of rs1965708 (*SFTPA2*, dominant) rs1136450 (*SFTPA1*, dominant) and rs2243639 (odds ratio of 1.62) they were considered protective (143). A similar approach, although restricted to genes encoding pulmonary collectins, was earlier proposed by Thomas et al. (144). They found 1 two-marker (A at *SFTPD* rs2243639 and 1A^1^ of *SFTPA2*), 2 three-marker [(T at rs721917, A at rs2243639 and 1A^1^) and (A at rs2243639, 1A^2^ of *SFTPA2* and 6A^4^ of *SFTPA1*) and 2 four-marker [(T at rs721917, A at rs2243639, 1A^1^ and 6A^3^ of *SFTPA1*) and (T at rs721917, A at rs2243639, 1A^2^ and 6A^4^)] haplotypes (144). Later, an association of *SFTPA2* 1A^1^ and *SFTPA1* 6A^4^ haplotypes was confirmed by Jo et al. (145) and Tsitoura et al. (146), respectively. Moreover, Tsitoura et al. (146) considered *SFTPA2* 1A^5^ variant as RDS risk factor as well while Jo et al. (145) suggested *SFTPA2* 1A^0^ protective of preterm neonates. Some seemingly contradictory data were published with regard to role of *SFTPA1* 6A^2^ and 6A^3^ variants in RDS. Several reports demonstrated 6A^2^ haplotype/homozygosity alone or in cooperation with other SNP/haplotypes of surfactant proteins-encoding genes as a risk factor. Ramet et al. (147) found 6A^2^ haplotype and its combination with 1A^0^ more frequent among RDS neonatal (mostly but not only preterm) cases, compared with controls. The 6A^3^ haplotype, especially when accompanied by *SFTPA2* 1A^1^ or 1A^2^ were deemed protective (147). Marttila et al. (148), in a study recruiting premature singletons and twins/multiples, observed higher frequency of both 6A^2^ haplotype and 6A^2^/6A^2^ genotype among affected singletons when accompanied by Thr/Thr homozygosity for *SFTPB* Ile131Thr polymorphism (rs1130866). Surprisingly, the discussed SP-A1 gene variants were underrepresented among twins/multiples with RDS when associated with Ile/Thr or Thr/Thr SP-B genotypes. Furthermore, it was found that the role of 6A^2^/Thr combination in singletons depends on gestational age: it seemed to increase the RDS risk when shorter and to protect when close to term (148). In another report, concerning twins born at GA 32-36 weeks, they observed 6A^2^ variant (also homozygous) less frequently in affected compared with unaffected babies. Moreover, that haplotype was noted to be associated with a higher birthweight (149). Earlier, Haataja et al. (150) demonstrated 6A^2^ and 6A^2^/1A^0^ combination more frequently among *SFTPB* Thr allele carrying preterms born at GA <32 weeks, diagnosed with RDS compared with corresponding controls. An opposite effect was observed for 6A^3^ and 6A^3^/1A^2^ variants (150). Some reports underlined certain differences depending not only on gestational age but also on ethnicity. Although Kala et al. (151) observed a protective association of homozygosity for 6A^3^ independently of those factors, the 1A^0^ allele effect was noticeable in Caucasians born at GA >28 weeks (including term neonates) but not in Afro-Americans or babies born at shorter GA. Again, a synergistic effect with the *SFTPB* polymorphism was noted (151). Later, Floros et al. (152) extended those findings demonstrating 6A^2^ and 1A^0^ variants as RDS risk factors when accompanied by specific *SFTPB* variants in preterm Caucasians as well as 6A^3^ one to be protective in Afro-Americans born at GA 31-35 weeks, in association with another SP-B gene polymorphism (152). Another issue considered by Haataja et al. (153) and Floros et al. (154) was parent-offspring transmission of genotypes and its association with phenotype. Haataja et al. (153) investigated that problem recruiting mother-father-preterm baby Finnish trios (neonates affected with RDS born at GA <37 weeks and unaffected born at GA <32 weeks, termed hypernormal). The 6A^2^/1A^0^ haplotype was found excessively transmitted to babies with RDS. On the other hand, decreased transmission of the 6A^2^ variant to unaffected preterms was observed (153). In the second mentioned work (154), families of various ethnicity were recruited. They confirmed a higher frequency of 6A^2^/1A^0^ haplotype and found a lower frequency of 6A^4^/1A^5^. Again, the 6A^3^ haplotype seemed protective for Afro-American but not Caucasian preterms. This study confirmed also some differences in distribution of susceptibility alleles, depending on gestational age (154).

Regarding RDS-associated SNP of genes for SP-A, as well as intra- or intergenic SNP combinations previously discussed, some single polymorphism relationships were described. Amatya et al. (143) demonstrated C allele at rs17886395 (*SFTPA2;* +271 G>C, Ala91Pro) to be protective from RDS. Earlier, Abuelhamed et al. (155) found A allele and A/A homozygosity for another *SFTPA2* polymorphism (+751 G>A) to be RDS risk factors. In the cited above study, Chang et al. (142) reported A/A genotype/A allele and C/C genotype/C allele related to rs1136451 (+186 A>G, Pro62Pro) and rs4253527 (+655 C>T, Arg219Trp) *SFTPA2* SNP, respectively, to be associated with neonatal RDS. Both mentioned variants corresponded to lower SP-A2 levels as well. Furthermore, analysis of haplotypes encompassing rs1136451, rs4253527 (*SFTPA2*), -18 A>C (5’UTR), rs1130866 (+1580 C>T, Thr131Ile) (*SFTPB*, encoding a hydrophobic lung surfactant component, SP-B), rs721917 and rs17885900 (*SFTPD*) led to the conclusion that ACACCA and ACACCG patterns may increase RDS risk in preterms while ACACTG seems to be protective (142). That finding again demonstrates the significance of inter- and intergenic associations.

Eguchi et al. (156) and Stevens et al. (157) observed lower SP-A levels in tracheal aspirates from babies with RDS in comparison with infants ventilated for other reasons. Furthermore, the latter investigation revealed no change in SP-A concentration within four days in non-survivors and a significant increase in survivors. Recently, Ghitoi et al. (158) found a significant correlation between expression of SP-A protein in the lung sections from fatal preterm RDS cases and bronchopneumonia. The association of lung SP-A expression and death from neonatal respiratory distress syndrome was however noticed over two decades ago by DeMello et al. (159, 160). Furthermore, low concentration of this lectin in the amniotic fluid was considered as predictor of fetal lung immaturity, and therefore RDS (although with low specificity) (120). Higher specificity was reached when SP-A levels were determined in cord sera from preterms (161, 162).

Another, already mentioned, severe clinical condition is bronchopulmonary dysplasia (BPD), affecting mainly preterm babies with extremely/very low birthweight, requiring mechanical ventilation and oxygen supplementation. It is often one of the consequences of RDS and invasive procedures used for treatment. Pavlovic et al. (163) investigated a variety of polymorphisms of surfactant protein genes in preterm infants suffering from BPD (majority of them were diagnosed with RDS as well). They suggested G- (*SFTPD* rs721917) -1A^2^ (*SFTPA2*) and C- (*SFTPD* rs17885900) -1A^2^ (*SFTPA2*) to be protective. Later, Vinod et al. (164) found no greater association of SP-D concentrations in serum (taken at 3^rd^ and 7^th^ days of life) with BPD at 36^th^ week of postmenstrual age in neonates born at GA ≤32 weeks. In a similar but not identical GA group (<32 weeks), Weber et al. (165) identified *SFTPA1* 6A^6^ haplotype as a BPD risk factor. Recently, De Luca et al. (132) observed lower concentrations of SP-D in BALF samples from extremely preterm newborns who developed that disease compared with those who did not or died with no diagnosis.

Neonatal RDS may also contribute to the development of chronic lung disease (CLD). Kotecha et al. (166) reported low activity (associated with presence of low oligomeric molecules) of SP-D in bronchoalveolar lavage fluid samples from preterm babies diagnosed with CLD. They furthermore observed lower SP-D expression at 1^st^ day of life in individuals who developed disease compared with those who did not (166).

Pulmonary morbidity may result from congenital diaphragmatic hernia (CDH) itself and related therapeutic procedures including mechanical ventilation. Marks et al. (167) noticed a marked increase of SP-D concentrations within the 1^st^ month in sera from infants who had more severe CHD. SP-D levels increased also in response to termination of extracorporeal membrane oxygenation (ECMO) as well as to diaphragm surgical repair. Those changes were suspected to reflect the lung injury (167).

Surfactant protein A is detectable in the blood-brain barrier and cerebrospinal fluid (CSF). Its elevated concentration, in comparison with reference group, was found in CSF from premature babies suffering from intraventricular haemorrhage. That was concluded to be associated with posthaemorrhagic CSF flow impairment (168). Recently, based on murine model, immunomodulatory role of SP-A in local and systemic neonatal neuroinflammation (intraventricular haemorrhage, hypoxic-ischemic encephalopathy, systemic sepsis) was demonstrated (169).

## 3 Clinical associations of ficolins in the neonate

### 3.1 Ficolin-1

Data concerning ficolin-1 in neonatal health and disease are still rather scarce. Its concentration in serum was demonstrated to increase with gestational age (79). Low levels of that protein were found associated with a requirement for mechanical ventilation and increased mortality in preterms with necrotising enterocolitis (170). Schlapbach et al. (171) reported also an association of high ficolin-1 with early-onset (but not late-onset) sepsis and its correlation with the absolute phagocyte count and immature/total neutrophil ratio. Later, Świerzko et al. (104) found higher median ficolin-1 concentrations in sera from babies suffering from perinatal infections who developed sepsis in comparison with healthy controls. A similar association was found with less severe infections, but no significant difference was noted between disease groups. Furthermore, heterozygosity for the rare *FCN1* gene mutation, rs148649884 (+6658 G>A, Ala218Thr) was reported in one case of neonatal systemic inflammatory response syndrome (SIRS) (104).

### 3.2 Ficolin-2

Ficolin-2 has been the most extensively investigated member of that family in relation to neonates. Several reports describe strong associations of its low concentrations in serum with prematurity and/or low birthweight (also independently of gestational age) (70, 79, 81, 172). However, Briana et al. (173) found no significant relationship between ficolin-2 levels in cord blood and intrauterine growth restriction in term newborns. Recently, Szala-Poździej et al. (174) reported associations of SNPs localised to the *FCN2* gene promoter with birthweight and/or gestational age in Polish preterm babies. Major alleles for rs3124952 (-986 A>G), rs7865453 (-64 A>C) and A/A homozygosity for both mentioned SNP were found less frequently among newborns with very low birthweight (VLBW, ≤1500 g) compared with the corresponding reference group. A similar relationship was observed for minor allele homozygosity (G/G) for rs17514136 (-4 A>G) polymorphism. That genotype seemed to protect from birth at GA <33 weeks as well. Moreover, reconstructed haplo- and diplotypes, including data concerning rs3124953 (-602 G>A) SNP were analysed in the context of GA and BW. The AGA (-986/-602/-64/-4) haplotype and AGAG/AGAG homozygosity were protective for VLBW. The GGCA haplotype had the opposite effect, independently of GA. Finally, the AGAG/GGAA diplotype was associated with both shorter (<33 weeks) GA and body mass at birth <1500 g (174). Earlier, Kilpatrick et al. (70) found a lower frequency of the A/A-A/A-A/A-A/A-A/A-C/C-G/G genotype [related to following *FCN2* SNP: rs3124952 (-986 A>G), rs3124953 (-602 G>A), rs3811140 (-557 A>G), rs7865453 (-64 A>C), rs17514136 (-4 A>G), rs17549193 (+6359 C>T, Thr236Met) and rs7851696 (+6424 G>T, Ala258Ser)] in preterm, compared with term neonates. Those findings seem to confirm a significant relationship between low ficolin-2 and prematurity as well as low BW. Furthermore, two *FCN2* SNP localised to 3’UTR: rs73664188 (T>C) and rs11103564 (T>C) were found to influence the risk of VLBW: the C allele of the first was found to increase the chance of BW <1500 g, while that of the second was protective in preterm newborns (71).

Low ficolin-2 concentration in cord serum was also found associated with a higher risk of perinatal infections, including sepsis (70, 81, 104). In those studies, data from consecutively taken samples (both term and preterm babies) were analysed. Low (<1 µg/ml) ficolin-2 levels were found significantly more often in disease groups (babies with perinatal infections who developed sepsis or with milder disease course) compared with infection-free controls (104). However, in another investigation, Schlapbach et al. (107) observed no significant difference between infants suffering from culture-proven sepsis and matched controls. Furthermore, no impact of the *FCN2* promoter (-64 A>C and -4 A>G) or exon 8 (+6359 C>T and +6424 G>T] SNPs on risk of perinatal sepsis (104) or the promoter (-986/-602/-64/-4) SNP on early-onset infection in preterms (174) was found. On the other hand, Świerzko et al. (71) demonstrated diplotypes GTTTGT/GGTCGT and GTTTGT/GGTCGA [corresponding to *FCN2* 3’UTR polymorphisms: rs7851696 (G>T), rs4521835 (T>G), rs73664188 (T>C), rs11103564 (T>C), rs11103565 (G>A) and rs6537959 (T>A)] to be risk factors for early onset infections (EOI) in preterm babies. The protective influence of ficolin-2 on certain perinatal infections was strongly supported from evidence by Fuijeda et al. (175). They demonstrated its contribution to opsonophagocytosis and killing of group B streptococci (GBS, a common agent of neonatal infections, including pneumonia, meningitis and sepsis) of III, V and VIII serotypes (175).

Recently, Gajek et al. (176), investigating the previously-described cohort of preterm babies (71, 174), observed significantly lower concentrations of ficolin-2 in cord serum samples (independently of *FCN2* promoter/3’UTR SNP) from individuals who developed RDS. Ficolin-2 concentrations were lower in babies who required intubation in the delivery room. Moreover, ficolin-2 levels correlated positively with Apgar score and inversely with duration of respiratory support and length of NICU stay. When GA did not exceed 32 weeks, ficolin-2 concentration partially distinguished those with and without RDS and differentiated between mild and moderate/severe disease (176).

### 3.3 Ficolin-3

Like other ficolins, ficolin-3 concentration increases with gestational age (77, 177), and correlates significantly with both GA and BW as well as with ficolin-2 (177). Low levels are associated with low birthweight independently of GA (177), but there was no relationship with IUGR in term babies (173). Schlapbach et al. (178) suggested that inherited deficiency of ficolin-3 may be responsible for necrotising enterocolitis as well as repeated staphylococcal skin infections (178). Michalski et al. (179) described a patient with severe *Streptococcus agalactiae* infection, but that individual had multiple immune defects so the contribution of ficolin-3 was uncertain. Additionally, for both those patients, no more severe infections were noted within 4-year and 8-year follow-ups, respectively (178, 179). Low ficolin-3 was found associated with neonatal sepsis by Świerzko et al. (104), and specifically Gram-positive sepsis by Schlapbach et al. (107). Although the median value determined for newborns with proven infections who had not developed sepsis did not differ from that of controls (104, 177), the frequency of low (<7.9 µg/ml) concentrations was significantly higher (104).

## 4 Concluding remarks

Collectins and ficolins create an universal group of pattern-recognising receptors having collectively broad specificity towards non-self or abnormal-self glycoconjugates (but not limited to saccharide structures). Their affinity to the target is enhanced by multimerization, enabling more efficient opsonisation or agglutination of pathogens. The majority is expressed mainly in hepatocytes and secreted into blood, but some (synthesized also within respiratory system) play an important role in local immunity (SP-A, SP-D, ficolin-3). CL-12 and ficolin-1 are produced by other cells and may be found as soluble or cell-associated forms. With the exception of SP-A and SP-D, they are capable of initiating complement activation, mostly via the lectin pathway, although CL-12 is known to initiate alternative and classical pathways. In this paper, we described the genetics, structure, and specificity of these collagen-related lectins, and reviewed their complex and multiple clinical associations in neonates. Their protective role or contribution to the pathogenesis, depend on a variety of variables, including genetic polymorphisms, gestational age, way of delivery, maternal/environmental (i.e. hospital/home) microflora, and so on.

## Author contributions

MC: Conceptualization, Supervision, Writing – original draft, Writing – review & editing. AS: Conceptualization, Writing – review & editing.
